# Rizzoli’s Osteoklast; Hegar’s Operation for Fibroid of the Uterus; Akystopeurastic

**Published:** 1878-10

**Authors:** N. Senn

**Affiliations:** Munich


### Correspondence.

### RIZZOLI'S OSTEOKLAST—HEGAR'S OPERATION FOR FIBROID OF THE UTERUS—AKYSTOPEURASTIC.

MUNICH, August 1, 1878.

*Editors Chicago Medical Journal and Examiner:*

In my last letter I described Ogsdon's operation for genu valgum, and reported a case operated upon by Prof. Von Nussbaum. The result, as in all previous cases, has been perfect. The limb was retained in its normal position with a straight splint without any difficulty; no fever followed, and the pain was inconsiderable. Passive motion was made twelve days after the operation, and repeated daily. Subcutaneous osteotomy, for correcting angularity of long bones, has been entirely abandoned here, and osteoklase with Rizzoli's instrument has been substituted. During this session Prof. Von Nussbaum has made numerous experiments with this instrument on cadavers; all of them have proven: 1, any of the long bones can be fractured; 2, the fracture takes place at the desired spot; 3, the operation produces a simple fracture, without any serious complications. To avoid injury to the vessels the fracture must always be made in the direction of the deformity, and the limb straightened gradually and carefully.

This instrument was used on a little girl with outward curvature of both legs. Both legs were fractured. The fracture took place with a loud snap. The limbs were placed in the natural position and a plaster-of-Paris dressing applied. No reaction took place—the patient made a favorable recovery.

In ovariotomy operations, the pedicle is secured with two or more stout catgut ligatures, and always left intra-peritoneal. If the pedicle is large it is tied in two or more parts, and to give additional security in preventing secondary hemorrhage, the large vessels are separately ligated with catgut at the cut end of the stump. No drainage tubes are employed. Of the three operations which I saw, all concerned patients, between 30 and 40 years of age; tumors, simple cysts; in one case extensive adhesions—they all recovered; only one presented slight evidences of peritonitis. Prof. Von Nussbaum believes that the peritoneum is not liable to inflammation, provided it is not exposed to infection, hence the importance of opening the peritoneal cavity under the antiseptic spray.

Another very important item he considers to be the employment of artificial heat during the operation. The room is thoroughly disinfected with carbolic spray an hour before the operation, and the temperature raised to 75° to 80° F., vessels with hot water are placed near the extremities and body of the patient, the carbolic acid solution for the spray and for cleansing the wound must also be warm.

As the peritoneum is one of the most active absorbing surfaces for infectious matter, all operations which necessitate its exposure should be carried out on strict antiseptic principles.

Hegar's operation of removing both ovaries for fibrous tumor of uterus has been performed here three times. The principle of the operation consists in anticipating the climateric period, and by arresting the physiological congestions of the organ, arrest the morbid process, and render the tumor amenable to absorption. One of these cases proved successful. The patient had been so much reduced by profuse hæmorrhages that she was confined to bed. The tumor was large, involving the greater part of the uterus. Both ovaries were removed, through a small incision in the linea alba. She recovered rapidly, and after the lapse of nearly a year the entire tumor had disappeared. I witnessed only the last operation. The patient was about 40 years of age. The tumor had existed for many years, causing, the last few months, serious loss of blood. She has suffered on several occasions attacks of severe pain, indicating the existence of previous pelvic peritonitis. Attempts had been made to promote the absorption of the tumor by the introduction of sponge-tents through the cervical canal. but were always followed by symptoms of inflammation, consequently they had to be abandoned. As a last resort it was decided to perform Hegar's operation, as the only chance to save her life. A small incision was made in the linea alba, half way between the pubes and umbilicus, where the upper margin of the tumor was found. The tumor was connected to surrounding parts by adhesions, and the peritoneum showed signs of recent inflammation. The incision was enlarged to the umbilicus, and the right ovary brought forward and removed, after ligating its pedicle firmly with catgut. The left ovary was found intimately connected with the surface of the tumor; in order to reach it a lateral incision had to be made. The whole ovary could only be removed after a very tedious dissection and application of numerous ligatures. The wound was closed with silver wire and catgut sutures, the whole operation having been conducted on strict antiseptic principles. The patient rallied from the operation, but died thirty-six hours after from peritonitis. What had rendered the prognosis very unfavorable in this case were the firm adhesions of the left ovary and the existence of recent pelvic inflammation.

Akystopeurastic is a term introduced by Middeldorpf for designating a new method for exploration. The instrument employed is a long, slender needle. It is applicable, in the first place, as the most reliable means for ascertaining the existence of death. The needle is thrust into the chest, between the fifth and sixth ribs, so as to come in contact with the heart, or enter its substance; if life is not extinct the end of the needle will move synchronous with the heart; if death has taken place this motion will not be observed. It is likewise an important adjunct for diagnosing dislocations. In a doubtful case of injury of the shoulder joint the needle can be pushed below the acromion process toward the glenoid cavity; if the bone is not dislocated, it will meet with resistance on part of the head and neck of humerus; if dislocation has taken place, it will enter easily and meet with no obstruction until it comes in contact with the glenoid cavity. Simple as it is, this method will prove to be a valuable aid in surgical diagnosis.

Chronic varicose ulcers are treated as follows: The ulcerated surface is made aseptic by the application of an 8 per cent. solution of chloride of zinc, and covered with wet borax lint and gutta-percha tissue. As soon as healthy granulations make their appearance the surface is covered with dry borax lint, without the impermeable covering. The result of this treatment has been very beneficial.

This week the present semester will close, and with it the most interesting surgical clinics of Prof. Von Nussbaum.

I have seen so many valuable things in his practice that it would be impossible to communicate them all within such a brief space. They can only be appreciated by those whose fortune it is to witness his operations.

As a practical teacher and original thinker he has certainly no equal.

In my next communication I will attempt to tell you something of the remaining teachers and of the merits of this school.

Very truly, N. Senn, M. D.

